# Supplementary material for: A feedback queueing network model for traffic signal control at intersections considering congestion propagation in dynamic stochastic environments
Source: PLoS One. 2025 Dec 2;20(12):e0337201. doi: 10.1371/journal.pone.0337201 (PMC12671815; doi:10.1371/journal.pone.0337201)
Supplement: Attachment — Submitted filename: Response to Reviewers.docx [file pone.0337201.s001.docx]

Response to Reviewers

Dear Editors and Reviewers:

Thank you for your letter and for the reviewers’ comments concerning our manuscript entitled “A Feedback Queueing Network Model for Traffic Signal Control at Intersections Considering Congestion Propagation in Dynamic Stochastic Environments” (ID: PONE-D-25-24915). Those comments are all valuable and very helpful for revising and improving our paper, as well as the important guiding significance to our researches. We have studied comments carefully and have made correction which we hope meet with approval.

1. General Response

We sincerely thank the Editor and Reviewers for their constructive feedback. All comments have been carefully addressed in the revised manuscript. All changes are highlighted in blue text in the "Revised Manuscript with Track Changes" file.

2. **Response to Reviewer #1:**

The paper presents a feedback queueing network model for traffic signal control, addressing congestion propagation in dynamic stochastic environments. The work is well-structured, methodologically sound, and offers contributions to traffic signal optimization. However, following comments need to be addressed:

**Comment 1**: This paper includes a substantial amount of literature, much of which dates back 20 years. While some of these works are classic controllers, the reviewers recommend updating the literature review with recent studies. In particular, the authors reviewed related works on reinforcement learning, which is good. However, the cited references are not representative enough. The following works should be considered and incorporated: (1)Su, Z. C., Chow, A. H., & Zhong, R. X. (2021). Adaptive network traffic control with an integrated model-based and data-driven approach and a decentralised solution method. Transportation Research Part C: Emerging Technologies, 128, 103154.

(2)Su, Z. C., Chow, A. H., Fang, C. L., Liang, E. M., & Zhong, R. X. (2023). Hierarchical control for stochastic network traffic with reinforcement learning. Transportation Research Part B: Methodological, 167, 196-216.

(3)Liang, E., Su, Z., Fang, C., & Zhong, R. (2022). Oam: An option-action reinforcement learning framework for universal multi-intersection control. In Proceedings of the AAAI Conference on Artificial Intelligence (Vol. 36, No. 4, pp. 4550-4558).

**The author’s answer**: Thank you very much for your valuable suggestions in our article. The seminal papers you recommended are indeed classic works in the field of traffic signal control. Due to an oversight on our part during the literature review, these works were initially overlooked. We have since carefully studied these seminal papers and found their content highly relevant to the research presented in this article, providing significant guiding significance for our work. Therefore, we have cited them in the manuscript. The references corresponding to these aforementioned papers are numbered 16, 18, and 36, respectively.

**Comment 2**: The assumptions (e.g., Poisson arrivals, state-dependent service rates) are critical to the model's validity. While the paper mentions these, a solid discussion is needed to justify their real-world applicability (e.g., deviations from Poisson arrivals during peak hours) would strengthen the work.

**The author’s answer**: We will be happy to edit the text further, based on helpful comments from the reviewers.

(1) Regarding the assumption of Poisson arrivals, we acknowledge that the applicability of this fundamental assumption was indeed overlooked during our research process. The paper assumed that vehicle arrivals follow a Poisson distribution, primarily following established practices in related works such as[1–6]. We fully recognize the limitations inherent in this assumption. In subsequent research, we plan to develop a queueing network model for intersections based on the Phase-type (PH) distribution. This is motivated by the theoretical capability of the PH distribution to approximate arbitrary arrival patterns, offering superior applicability. Furthermore, the PH distribution is closely related to the exponential distribution; indeed, it serves as a generalization and extension of the exponential distribution. Therefore, developing an intersection queueing network model based on the PH distribution appears highly feasible. The specific modifications in the manuscript are: “*As rightly pointed out by the reviewer, the assumption of Poisson arrivals employed in this paper may deviate from actual traffic conditions. Deviations may particularly occur during peak hours or under the influence of traffic signals, making it difficult to guarantee that traffic flow arrivals strictly adhere to a Poisson distribution. The paper assumed that vehicle arrivals follow a Poisson distribution, primarily following established practices in related works such as [20,46,47,49,60,75]. While we fully acknowledge the limitations of this assumption, it remains fundamental to our current modeling approach and cannot be fundamentally altered within the scope of this study in the short term. In subsequent research, we plan to develop a queueing network model for intersections based on the Phase-type (PH) distribution. This is motivated by the theoretical capability of the PH distribution to approximate arbitrary arrival patterns, offering superior applicability. Furthermore, the PH distribution is closely related to the exponential distribution; indeed, it serves as a generalization and extension of the exponential distribution. Therefore, developing an intersection queueing network model based on the PH distribution appears highly feasible*.” **(Page 53, Lines 880-890)**.

(2) Regarding state-dependent service rates, the term "state-dependent service rate" used in this paper refers to the phenomenon where the road service rate changes according to the level of traffic congestion. This is a common phenomenon, most notably manifested by a significant decrease in road capacity when traffic becomes congested. In our previous work, we discussed state-dependent service rates [7]. The specific modifications in the manuscript are: “*State-dependent service rate refers to the phenomenon where the service rate of a road facility varies with the number of vehicles on the road. A prominent example is that when the number of vehicles is low, the road capacity is relatively high, while during congestion, the road capacity significantly decreases. State-dependent service rates have been studied in depth in our previous work [56]*.” **(Page 15, Lines 267-270)**.

**Comment 3**: The rolling optimization strategy’s dependency on accurate demand prediction (Step 2, Algorithm 2) is a potential limitation. How does the model perform with prediction errors?

**The author’s answer**: Thank you for your careful review and valuable suggestions. As rightly noted by you, the rolling optimization strategy's dependency on accurate demand prediction is indeed a potential limitation. We acknowledge that robustness analysis against prediction errors was not sufficiently addressed in the original manuscript. In response to this concern: (1) Foundation of demand prediction: Our approach relies on established short-term traffic demand forecasting methods which typically achieve high prediction accuracy for near-future horizons; (2) Error mitigation mechanism: The rolling optimization adjusts signal control parameters once per cycle. Consequently, prediction errors within a single cycle exert negligible impact (<1% performance degradation) on overall control effectiveness across extended operation periods (e.g., 1 hour), as demonstrated by sensitivity tests where ±20% demand perturbations in individual cycles resulted in less than 1% deviation in system-wide delay metrics. These findings collectively indicate the model's inherent resilience to transient prediction inaccuracies.

**Comment 4**: The authors are encouraged to discuss the scalability of the proposed controller to network level with multi-intersections.

**The author’s answer**: Thank you for your careful review and valuable suggestions. We have augmented the discussion on this aspect in the revised manuscript. The specific modifications in the manuscript are:” *Following the aforementioned methodology, an intersection queuing network model can be constructed, abstracting signalized intersections into a network of interconnected queuing nodes. Consistent with this framework, multi-intersection clusters can likewise be represented as queuing networks composed of multiple nodes. Consequently, the queuing network model for intersection clusters established in this study can be decomposed into multiple isolated intersections, enabling parallel optimization of signal timing plans for clustered intersections.*

*Furthermore, our prior research [56] has demonstrated that* *the computational complexity of the recursive algorithm for solving the intersection-cluster queuing network model is O(N^2^ ∆T)+O(N∆T), where N denotes the number of intersection service facilities and ∆T the number of discrete time intervals. When addressing short-term horizons (e.g., 10-minute periods), the transition probabilities can be assumed constant, further reducing complexity to O(T)+O(NT). This low-complexity characteristic endows the algorithm with significant potential for large-scale network applications.*” **(Page 25-26, Lines 478-487).**

**3. Response to Reviewer #2**:

**Comment 1**: Rewrite the introduction to clearly define the research problem and how the proposed method closes the gap.

**The author’s answer**: We sincerely appreciate the reviewer's meticulous evaluation and highly valuable suggestions. In response to the reviewer's comments, we have restructured the abstract to further refine the research focus and contributions of this work.

The specific modifications in the manuscript are:” *The intersection traffic flow model is the basis of the MPC. Hence, accurately modeling the intersection is of great importance for the development of effective signal control strategies. A vast number of models have been put forward. According to the level of details, these models can be classified into microscopic, mesoscopic, and macroscopic models. Microscopic models could capture the system's details but require extensive calibration work and colossal computation time. Additionally, microscopic models cannot provide a direct input-output relationship. Therefore, they are relatively better suited to offline traffic simulations [33]. As for macroscopic models, traffic dynamics is described by the mainstream deterministic traffic flow theory models, e.g., the kinematic wave model (KWM) [34–36], cell transmission model (CTM) [37], and METANET model [38]. Macroscopic models are computationally fast and only require simple inputs. However, they lack the capability of adequately modeling the stochastic nature of traffic [39], especially for the real-time MPC signal control, which has been more and more crucial for real-time traffic signal control in order to respond to natural stochastic variations in traffic[40,41]. Therefore, developing a model capable of simultaneously achieving computational efficiency and strong representational capacity holds significant importance for enhancing the effectiveness of adaptive traffic signal control.*

*In this paper, queuing theory is leveraged in an attempt to achieve the aforementioned objectives. As one of the most popular mesoscopic models, the queuing model has been used to formulate road traffic flow [20–22,42–59]. Compared with macroscopic models, the queuing model can better study stochastic service systems. Although the queueing model is not more accurate than the corresponding discrete-event simulation, it is typically preferred because it may provide exact solutions, allow structural insights, and is faster than the simulation. Therefore, this paper applies the queueing theory to accurately model signalized intersections in dynamic stochastic environments.*

*However, signalized intersection is a complex dynamic random service system. In addition to the inherent randomness of intersection, the traffic demand and path matrix between facilities are time-varying. Meanwhile, the service ability depends on the system state, i.e., traffic congestion will decrease service ability. Most importantly, it is difficult to capture the Congestion Propagation in Dynamic Stochastic Environments (CPDSE) among different facilities of the intersection. To address the inherent randomness in intersections, some queueing models or shockwave models used the exponential or normal distributions to describe the vehicle arrival-interval and service-time random variables [1,27,60]. To account for the decrease in service ability caused by congestion, the linear or exponential functions were applied in CTM or METANET models to formulate the state-dependent velocity [61,62]. To describe the traffic dynamics of intersections, most traffic flow models considered the time-varying traffic demand and path matrix. To capture the congestion propagation phenomenon in deterministic traffic environments, CTM models have been developed for signalized intersections[63,61]. The existing mesoscopic traffic flow models consider some items in the system’s inherent randomness, state-dependent service ability, time-varying traffic demand and path matrix, and congestion propagation (see the literature review for details), but lack a comprehensive consideration of these characteristics because of the complexity of CPDSE.*

*In this paper, we identify the fluid queuing network model as a suitable methodology to formulate those aforementioned dynamic, randomness, state dependence, and congestion propagation for signalized intersections with real-time MPC traffic control. To the best of our knowledge, no study simultaneously models these essential characteristics to quickly and accurately formulate the dynamic performance of signalized intersections. We focus on an isolated intersection which is easily generalized to multiple intersections using the corresponding network topology. We divide the intersection into three categories of facilities and model each facility as a queue. A feedback fluid queuing network model is then developed to consider the CPDSE through a succession of facilities where the random traffic demand and path matrix are time-varying and the random service ability is state-dependent.*” **(Page 3-4, Lines 50-88).**

**Comment 2**: Although Table 2 is helpful, several symbols, such as , are referenced before they are formally defined. To enhance readability, include a clear summary of the notation used in the main text before it is first used.

**The author’s answer**: We sincerely appreciate the reviewer's meticulous assessment and constructive feedback. Upon your reminder, we recognize the issues regarding symbol definitions in the original manuscript. To ensure all symbols are clearly defined prior to their first occurrence, we have: (1) Relocated Table 2 to the beginning of the Preparations section; (2) Systematically reviewed critical notations throughout the paper and augmented their definitions in Table 2. The specific modifications can be found in Page 10, Lines 193-194.

**Comment 3**: The use of continuous ensemble averages ( (t) ∈ [0, T]) for inherently discrete traffic flows may limit accuracy in high-congestion scenarios. Assumption that arrival rates follow a non-homogeneous Poisson process may not capture sporadic or correlated traffic. Justify the approximation error and explain whether it applies to real-time adaptive systems.

**The author’s answer**: We sincerely appreciate the reviewer's meticulous evaluation and highly valuable suggestions. **(1) Rationale for continuous ensemble averaging**. The adoption of continuous ensemble averaging ( (t) ∈ [0, T]) for inherently discrete traffic flows stems from the necessity to accommodate dynamic traffic demand variations over extended time horizons. This framework enables explicit incorporation of time-varying demand patterns into our model formulation. **(2) Justification for non-homogeneous Poisson process**. The assumption of non-homogeneous Poisson arrivals follows established methodologies pioneered by Osorio[1–6]. The specific modifications in the manuscript are: “*As rightly pointed out by the reviewer, the assumption of Poisson arrivals employed in this paper may deviate from actual traffic conditions. Deviations may particularly occur during peak hours or under the influence of traffic signals, making it difficult to guarantee that traffic flow arrivals strictly adhere to a Poisson distribution. The paper assumed that vehicle arrivals follow a Poisson distribution, primarily following established practices in related works such as [20,46,47,49,60,75]. While we fully acknowledge the limitations of this assumption, it remains fundamental to our current modeling approach and cannot be fundamentally altered within the scope of this study in the short term. In subsequent research, we plan to develop a queueing network model for intersections based on the Phase-type (PH) distribution. This is motivated by the theoretical capability of the PH distribution to approximate arbitrary arrival patterns, offering superior applicability. Furthermore, the PH distribution is closely related to the exponential distribution; indeed, it serves as a generalization and extension of the exponential distribution. Therefore, developing an intersection queueing network model based on the PH distribution appears highly feasible*.” **(Page 53-54, Lines 880-890)**. While we acknowledge that queuing theory, as a mesoscopic modeling paradigm, inherently cannot capture sporadic platoons or vehicle-to-vehicle correlations (as rightly noted), its adoption reflects a deliberate trade-off between analytical tractability and computational efficiency - a core motivation explicitly stated in our Introduction. The specific modifications in the manuscript are: “*As for macroscopic models, traffic dynamics is described by the mainstream deterministic traffic flow theory models, e.g., the kinematic wave model (KWM) [34–36], cell transmission model (CTM) [37], and METANET model [38]. Macroscopic models are computationally fast and only require simple inputs. However, they lack the capability of adequately modeling the stochastic nature of traffic [39], especially for the real-time MPC signal control, which has been more and more crucial for real-time traffic signal control in order to respond to natural stochastic variations in traffic [40,41]. Therefore, developing a model capable of simultaneously achieving computational efficiency and strong representational capacity holds significant importance for enhancing the effectiveness of adaptive traffic signal control. In this paper, queuing theory is leveraged in an attempt to achieve the aforementioned objectives. As one of the most popular mesoscopic models, the queuing model has been used to formulate road traffic flow [20–22,42–59]. Compared with macroscopic models, the queuing model can better study stochastic service systems. Although the queueing model is not more accurate than the corresponding discrete-event simulation, it is typically preferred because it may provide exact solutions, allow structural insights, and is faster than the simulation. Therefore, this paper applies the queueing theory to accurately model signalized intersections in dynamic stochastic environments.*”. **(Page 3, Lines 50-61)**. Crucially, the model maintains prediction errors below 10% compared to microscopic simulations, which we deem acceptable for practical adaptive control implementations. The specific modifications in the manuscript are: “*Fig 9-Fig 11 shows that the difference between the simulation model results and those of the proposed model is tiny regardless of the variance in traffic intensity. Compared with the mean result of the 200 simulations, the average absolute error is 0.5152 vehicles, and the average relative error is 6.43%. Fig 9-Fig 11 shows that the proposed model can capture the dynamic performance measures of the intersection accurately. Therefore, the proposed Mt/G(x)/C/C feedback queueing network model accurately describes the dynamic performance of the intersection. It should be noted that each simulation takes nearly 0.5 hours. The proposed model only takes around 0.2 seconds, although the approach's time step is set to 1 second, and the period [17:00, 18:00) is divided into 3600 segments. This indicates that the proposed recursive algorithm in Algorithm 1 has high computational efficiency for the intersection queue network system. It is promising to solve large-scale problems such as 200 intersections which is our ongoing work*”. **(Page 35, Lines 644-652). (3)** **Real-time applicability**. Our prior work [7] demonstrates that the computational complexity of the recursive algorithm for solving the intersection-cluster queuing network model is O(N2 ∆T)+O(N∆T), where N denotes the number of intersection service facilities and ∆T the number of discrete time intervals. When addressing short-term horizons (e.g., 10-minute periods), the transition probabilities can be assumed constant, further reducing complexity to O(T)+O(NT). This low-complexity characteristic endows the algorithm with significant potential for large-scale network applications. This efficiency profile, combined with modern edge-cloud computing infrastructure, confirms the algorithm's viability for real-time adaptive control in single intersection and scalable deployment to intersection clusters. The specific modifications in the manuscript are:” *Following the aforementioned methodology, an intersection queuing network model can be constructed, abstracting signalized intersections into a network of interconnected queuing nodes. Consistent with this framework, multi-intersection clusters can likewise be represented as queuing networks composed of multiple nodes. Consequently, the queuing network model for intersection clusters established in this study can be decomposed into multiple isolated intersections, enabling parallel optimization of signal timing plans for clustered intersections.*

*Furthermore, our prior research [56] has demonstrated that the computational complexity of the recursive algorithm for solving the intersection-cluster queuing network model is O(N^2^ ∆T)+O(N∆T), where N denotes the number of intersection service facilities and ∆T the number of discrete time intervals. When addressing short-term horizons (e.g., 10-minute periods), the transition probabilities can be assumed constant, further reducing complexity to O(T)+O(NT). This low-complexity characteristic endows the algorithm with significant potential for large-scale network applications.*” **(Page 25-26, Lines478-487).**

**Comment 4**: The model for congestion propagation relies on artificial nodes and blocking probabilities that may not fully capture driver reaction times, lane-switching, or partial blockages. Discuss whether the model is extendable to mixed traffic environments with human and autonomous vehicles.

**The author’s answer**: We sincerely appreciate the reviewer's meticulous assessment and constructive feedback. As rightly noted, the traffic flow model in this study is developed based on queuing theory. Being a mesoscopic modeling paradigm, queuing theory inherently cannot capture microscopic behaviors such as driver reaction times, lane-switching maneuvers, or partial blockages. This limitation stems from our fundamental design objective: to strike a deliberate trade-off between computational efficiency and modeling accuracy. Capturing such microscopic behaviors would inevitably introduce significant model complexity, substantially increasing computational demands and thereby compromising the model's applicability to large-scale networks. The specific modifications in the manuscript are: “*As for macroscopic models, traffic dynamics is described by the mainstream deterministic traffic flow theory models, e.g., the kinematic wave model (KWM) [34–36], cell transmission model (CTM) [37], and METANET model [38]. Macroscopic models are computationally fast and only require simple inputs. However, they lack the capability of adequately modeling the stochastic nature of traffic [39], especially for the real-time MPC signal control, which has been more and more crucial for real-time traffic signal control in order to respond to natural stochastic variations in traffic[40,41]. Therefore, developing a model capable of simultaneously achieving computational efficiency and strong representational capacity holds significant importance for enhancing the effectiveness of adaptive traffic signal control. In this paper, queuing theory is leveraged in an attempt to achieve the aforementioned objectives. As one of the most popular mesoscopic models, the queuing model has been used to formulate road traffic flow [20–22,42–59]. Compared with macroscopic models, the queuing model can better study stochastic service systems. Although the queueing model is not more accurate than the corresponding discrete-event simulation, it is typically preferred because it may provide exact solutions, allow structural insights, and is faster than the simulation. Therefore, this paper applies the queueing theory to accurately model signalized intersections in dynamic stochastic environments.*”. **(Page 3, Lines 50-61)**. Moreover, the proposed model is extensible to mixed traffic environments comprising both human-driven vehicles (HVs) and automated vehicles (AVs). Due to distinct operational characteristics between HVs and AVs, their driving behaviors exhibit fundamental differences. Within the queuing theory framework, such heterogeneity can be accommodated through a multi-class queuing system, where distinct vehicle types are treated as separate customer classes with differentiated service parameters (e.g., service rates, headway distributions). This enables the development of a unified queuing network model capable of capturing mixed traffic dynamics. We have delineated this extension strategy in the Future Work section of the paper. The specific modifications in the manuscript are: “*Meanwhile, traffic control strategies under the proposed model is extensible to mixed traffic environments comprising both human-driven vehicles (HVs) and automated vehicles (AVs). Due to distinct driving behaviors (characterized by car-following models) between HVs and automated vehicles AVs, their traffic flow characteristics exhibit significant differences. To model mixed traffic environments with coexisting HVs and AVs, we propose implementing a multi-class queuing system. Within this framework: (1) Vehicle-type heterogeneity is accommodated through differentiated service characteristics. (2) Driving behavior disparities are captured via class-specific service parameters. Furthermore, the service rate in queuing systems depends on the average speed of vehicles within the system. Consequently, we will: (1) Investigate fundamental diagrams under mixed traffic conditions. (2) Calibrate average travel speeds for queuing model parameterization. Subsequently, by treating distinct vehicle types as multiple customer classes in the feedback queuing network model, a multi-class queuing network model for signalized intersections will be developed to represent mixed traffic dynamics. Developing and refining multi-class queuing theory to characterize mixed traffic flow constitutes a primary research direction for our future work.*” **(Page 52-53, Lines 861-872).**

**Comment 5**: The only benchmark used is Synchro. Although practical, it is not a state-of-the-art real-time signal control system. Compare with other recent MPC-based or AI-driven adaptive control methods such as SURTRAC, MARLIN-ATSC, or deep reinforcement learning approaches.

**The author’s answer**: We sincerely appreciate the reviewer's meticulous assessment and constructive feedback. We have added a dedicated section titled "Comparison with Existing Methods". Within this section, comparative analysis with Osorio's[2] approach demonstrates that our optimization method incorporating congestion propagation achieves superior control performance across diverse demand scenarios. The specific modifications in the manuscript are: “*The proposed method in this paper shares certain similarities with the approach by Osorio[60].Therefore, a comparison with their method is conducted to further demonstrate the effectiveness of our model. Osorio[60] employed an analytical* $M(t)/ M/1/C$ *queuing network model to characterize traffic flow at intersections. However, their model abstracts the roadway link as a queuing network with a single server, fails to distinguish and characterize the traffic flow properties of different facilities, and does not account for the state-dependent nature of facility service capacity.*

*The optimization results obtained using the method proposed by Osorio[60] under different demand scenarios and initial state conditions are presented in Fig 13.*

|  |  |
| --- | --- |
| *(a) The existing method* | *(b) The proposed method* |

*Fig 13. Optimal average delay time of the proposed model with rolling optimization strategy*

*Compared to the method proposed by Osorio[60], our approach consistently achieves a better optimal average vehicle delay time, regardless of the initial system state or traffic demand scenario. This is because the method by Osorio[60] fails to account for the state-dependent service rate. It invariably provides an overly optimistic estimate of the intersection system's output rate, leading to excessive congestion in some facilities and consequently increasing vehicle delay times.*

*Under low traffic demand conditions, the optimal average vehicle delay times obtained by our proposed method and the method by Osorio[60] are very similar. This occurs because the probability of system congestion is low when traffic demand is small. Consequently, the assessments of the system output rate by both methods are relatively close, yielding similar results.*

*Fig 14.* *The extent of improvement achieved by the proposed* *method over existing methods*

*Compared to the method proposed by Osorio[60], the approach presented in this paper achieves further improvements in the optimal average delay time at intersections. The magnitude of these improvements is illustrated in Fig 14. The results demonstrate that compared to the method proposed by Osorio[60], the approach presented in this paper further reduces the optimal average delay time at intersections. The improvement is most significant under moderate traffic conditions, reaching a maximum reduction of 34.22%. This is because during moderate traffic conditions, the system experiences occasional congestion. Our model, which incorporates state-dependency, captures the system's dynamic performance more accurately, thereby enabling the optimization method to yield superior signal timing plans. Furthermore, the aforementioned results also indicate that, relative to the method by Osorio et al., our proposed method achieves further improvements in the optimal average intersection delay time across various traffic demands and initial states, albeit to varying degrees.*” **(Page 42-44, Lines 730-756).**

**Comment 6**: Include sensitivity analysis over more cities and intersection types or highlight generalizability limitations.

**The author’s answer**: We sincerely appreciate your insightful suggestions regarding model generalizability. While the relevant data from additional cities has not yet been obtained, we are actively acquiring foundational datasets from multiple urban areas to conduct this analysis. Additionally, we have incorporated a comprehensive discussion of the model's limitations within the Conclusion section. The specific modifications in the manuscript are: “*It should be noted that the proposed model still exhibits several limitations: (1)Inflow/outflow dynamics: The current formulation inadequately captures nuanced traffic flow interactions between adjacent road segments, requiring further refinement in modeling lane-specific inflow-outflow patterns.(2) Poisson arrival assumption: The premise of Poisson-distributed arrivals may not hold under signal-controlled conditions where vehicle platooning effects dominate, necessitating future relaxation of this constraint. (3) Scope restriction: The exclusive focus on signalized intersections leaves unsignalized intersections (e.g., roundabouts, stop-controlled junctions) unmodeled. Consequently, large-scale real-world applications demand extensions to characterize uncontrolled intersection dynamics.*” **(Page 53, Lines 873-879).**

**References**

1.	Osorio C, Nanduri K. Energy-Efficient Urban Traffic Management: A Microscopic Simulation-Based Approach. Transp Sci. 2015;49: 637–651. doi:10.1287/trsc.2014.0554

2.	Osorio C, Yamani J. Analytical and Scalable Analysis of Transient Tandem Markovian Finite Capacity Queueing Networks. Transp Sci. 2017;51: 823–840. doi:10.1287/trsc.2015.0629

3.	Osorio C, Flötteröd G, Zhang C. A Metamodel Simulation-based Optimization Approach for the Efficient Calibration of Stochastic Traffic Simulators. Transp Res Procedia. 2015;6: 213–223.

4.	Osorio C, Bierlaire M. A surrogate model for traffic optimization of congested networks : an analytic queueing network approach. Environ Eng. 2009; 1–23.

5.	Osorio C, Bierlaire M. A simulation-based optimization framework for urban transportation problems. Oper Res. 2013;61: 1333–1345. doi:10.1287/opre.2013.1226

6.	Chong L, Osorio C. A simulation-based optimization algorithm for dynamic large-scale urban transportation problems. Transp Sci. 2018;52: 637–656.

7.	Hu L, Zhao B, Zhu J, Jiang Y. Two time-varying and state-dependent fluid queuing models for traffic circulation systems. Eur J Oper Res. 2019;275: 997–1019.

Should you require further revisions, we welcome additional feedback.

Yours sincerely,

Bin Zhao

Email:1220220025@mail.xhu.edu.cn

August 5, 2025
