# Supplementary material for: A feedback queueing network model for traffic signal control at intersections considering congestion propagation in dynamic stochastic environments
Source: PLoS One. 2025 Dec 2;20(12):e0337201. doi: 10.1371/journal.pone.0337201 (PMC12671815; doi:10.1371/journal.pone.0337201)
Supplement: Attachment — Submitted filename: Response to Reviewers .docx [file pone.0337201.s002.docx]

Response to Reviewers

Dear Editors and Reviewers:

Thank you for your letter and for the reviewers’ comments concerning our manuscript entitled “A Feedback Queueing Network Model for Traffic Signal Control at Intersections Considering Congestion Propagation in Dynamic Stochastic Environments” (ID: PONE-D-25-24915). These comments are all valuable and very helpful for revising and improving our paper, and they also provide important guidance for our future research. We have studied comments carefully and have made correction which we hope meet with approval.

1. **Response to Reviewer #1:**

**Comment 1**: All my comments are well addressed. Good work.

**The author’s answer**: We sincerely appreciate your recognition of our work. Your valuable feedback has been instrumental in enhancing the quality of our paper. Should you have any further suggestions or comments, please do not hesitate to let us know, and we will continue to diligently examine them.

**2. Response to Reviewer #2**:

**Comment 1**:

(1) For Poisson arrivals, the authors added a discussion (page 53, lines 880–890) in which they acknowledged limitations (e.g. deviations in peak hours or under signal influence) and cited related works. They also proposed the future use of phase-type (PH) distributions for a better approximation. For state-dependent service rates, they provided a definition and justification (page 15, lines 267–270), referencing their previous work [56]. This strengthens the discussion, but the response defers fundamental changes to future research, which is reasonable but highlights ongoing limitations.

(2) The authors justify the use of continuous averages for handling time-varying demand (see page 3, lines 50-61). They acknowledge the limitations of the Poisson assumption and propose PH distributions for future work ( page 53-54, lines 880-890). Approximation error is justified by prediction errors vs. simulations (page 35, lines 644-652), with computational efficiency noted (0.2s vs. 0.5 hours for simulations). Real-time applicability is discussed via low complexity (see page 25-26, lines 478-487), confirming viability for adaptive systems. However, the response emphasises trade-offs (efficiency vs. accuracy) rather than fully resolving microscopic limitations.

(3) The authors added a "Comparison with Existing Methods" section (Page 42-44, Lines 730-756), comparing to Osorio [60] (an analytical queuing model), showing superior performance (up to 34.22% delay reduction under moderate demand) with figures (Fig 13-14). However, this does not directly address the suggested benchmarks (SURTRAC, MARLIN-ATSC, or DRL approaches). Osorio is relevant but not state-of-the-art AI/MPC; the response focuses on one method, so it partially addresses the spirit but misses the specific recommendations.

**The author’s answer**:

(1) For Poisson arrivals, we agree with the reviewer that deferring fundamental changes—such as adopting Phase-Type (PH) distributions—to future work underscores a known limitation of our current model. The primary focus of this study was to establish a computationally efficient and tractable foundation within the M_t_/G(x)/C/C framework that can explicitly capture congestion propagation and state-dependency – aspects often oversimplified in prior analytical models. We believe that successfully integrating these complex dynamics, even under the current assumptions, constitutes a significant step forward. The proposed use of PH distributions is a recognized and promising avenue for enhancing model fidelity, and we have now made this limitation and future direction more explicit in the revised manuscript (**Page 55-56, Lines 926-936**).

(2) Thank you for your insightful comment. As you rightly pointed out, our model emphasizes the trade-off between efficiency and accuracy. We would like to clarify that our proposed model is a mesoscopic, fluid-based approach. Its core strength lies in bridging the gap between highly accurate but computationally prohibitive microscopic simulations and efficient but often oversimplified macroscopic models. While it does not resolve vehicle-level dynamics (a microscopic limitation), it provides a principled and fast analytical tool for system-level performance evaluation and optimization, which represents a key requirement for real-time adaptive signal control. The significant computational advantage makes large-scale or repeated optimization feasible, constituting a primary contribution of this work. We have added a statement in the conclusion to better frame this trade-off as a conscious design choice for our intended application.

(3) Thank you for your constructive feedback. In response, during this revision cycle we have implemented both SURTRAC and MARLIN-ATSC, and conducted a comprehensive comparative analysis between the proposed method and these two benchmark approaches using the case study presented in this paper. Therefore, a new section titled "Comparison with Benchmark Methods" has been added to this paper.

The specific modifications in the manuscript are:”

***(2) Comparison with benchmark methods***

*In current research on traffic signal control, the SURTRAC* [1] *method and the MARLIN-ATSC* [2] *method represent landmark research outcomes. Therefore, this paper also conducts a comparative analysis with the aforementioned benchmark methods.*

*SURTRAC*[1]*, developed by the research team at Carnegie Mellon University, is a decentralized real-time traffic signal control method with scalability at its core. Its fundamental principle lies in decomposing network-wide control into autonomous decision-making at individual intersections. Each intersection generates local signal schedules through rolling-horizon optimization and achieves predictive coordination with adjacent intersections via the sharing of lightweight "itinerary plans." The advantages of this method include exceptional computational efficiency, robust real-time adaptability, high robustness, and demonstrated effectiveness validated through field deployments (e.g., successfully reducing delays by over 40% in Pittsburgh). However, it is inherently a local optimization approach, which may not achieve global optimality, and its coordination efficacy relies on a stable communication network while being sensitive to the accuracy of sensor data. Despite these limitations, it remains a benchmark solution that successfully applies intelligent optimization theory to smart traffic management, combining practical utility with significant impact.*

*MARLIN-ATSC*[2] *is a pioneering multi-agent reinforcement learning framework for traffic control, developed by the research team at the University of Toronto. Its core concept enables each intersection agent to autonomously learn signal control policies through interaction with the environment, utilizing unique coordination mechanisms such as ICQ and DCQ to resolve competition and dependency issues among intersections. The method's prominent advantages lie in its powerful data-driven learning capability, which allows it to adapt to complex dynamic environments without predefined traffic models, and its significant potential to discover coordination strategies beyond traditional approaches. However, it is constrained by key challenges including prolonged training times due to high sample complexity, poor interpretability caused by the black-box nature of deep neural networks, and difficulties in ensuring the safety and stability of learned policies in real-world applications. These factors collectively limit its current potential for large-scale practical deployment and application.*

*Based on References* [1] *and* [2]*, this study implemented both the SURTRAC and MARLIN-ATSC traffic signal control methods using Python to serve as benchmarks for comparison with the proposed method. The traffic demand and road network configurations remained consistent with those presented earlier in Tables 4 and Tables 5. Additionally, the initial system state was set at 0.3 times of the facility capacity. The results of the case study validation are summarized in Table 7.*

*Table 7 Performance Comparison between the Proposed Method, SURTRAC, and MARLIN-ATSC*

| *Performance Indicators* | *Proposed Method* | *SURTRAC* | *MARLIN-ATSC* |
| --- | --- | --- | --- |
| *Average Delay Time (s)* | *107.74* | *112.29* | *103.62* |
| *Computation Time (s)* | *48.1* | *63.5* | *82.6* |
| *Interpretability* | *High* | *Moderate* | *Low* |
| *Scalability* | *High* | *High* | *Moderate* |
| *Data Dependency* | *Low* | *Moderate* | *Extremely high* |

*Based on the comparative analysis and the data presented in Table 7, the proposed model demonstrates distinct and compelling advantages when evaluated against the two benchmarks, SURTRAC and MARLIN-ATSC.*

*In terms of control performance, both the proposed method and MARLIN-ATSC delivered excellent results, with very close average delay times (107.74 s and 103.62 s, respectively), and both outperformed SURTRAC (112.29 s). This indicates that all three methods are effective for intersection optimization, with MARLIN-ATSC's powerful data-driven learning capability enabling it to achieve the lowest delay under specific conditions.*

*However, the proposed model holds significant advantages in computational efficiency, interpretability, and practical applicability. As shown in Table 7, the computation time of the proposed method (48.1 s) is substantially lower than that of both SURTRAC (63.5 s) and MARLIN-ATSC (82.6 s), highlighting its superior potential for real-time applications. More importantly, the proposed model, grounded in explicit queuing theory, offers high interpretability, making its decision-making process transparent and trustworthy. In contrast, the "black-box" nature of MARLIN-ATSC's deep neural networks results in low interpretability, which hinders debugging and trust in practical deployments. Furthermore, the proposed model exhibits low data dependency, requiring only conventional traffic flow data. MARLIN-ATSC, conversely, relies on an extremely high volume and quality of training data, which severely constrains its applicability.*

*In summary, while MARLIN-ATSC can achieve marginally superior control performance when data is abundant, the proposed model offers a more balanced profile across computational efficiency, interpretability, and low data dependency. Compared to SURTRAC, the proposed model's explicit incorporation of congestion propagation and state-dependent characteristics, as opposed to their implicit handling within the prediction process, leads to slightly better performance. In conclusion, this research presents a promising solution for real-time traffic signal control that is not only effective and reliable but also highly practical for real-world deployment.*” **(Page 44-46, Lines 758-805).**

**References**

1.	Smith S, Barlow G. SURTRAC: Scalable Urban Traffic Control. Transportation Research Board Meeting. 2013.

2.	El-Tantawy S, Abdulhai B. Multi-Agent Reinforcement Learning for Integrated Network of Adaptive Traffic Signal Controllers (MARLIN-ATSC). International IEEE Conference on Intelligent Transportation Systems. 2012.

Should you require further revisions, we welcome additional feedback.

Yours sincerely,

Bin Zhao

Email: dalouluo@svtcc.edu.cn

October 22, 2025
